# Neurological symptoms, manifestations, and complications associated with severe acute respiratory syndrome coronavirus 2 (SARS-CoV-2) and coronavirus disease 19 (COVID-19)

**DOI:** 10.1007/s00415-021-10406-y

**Published:** 2021-01-23

**Authors:** Biyan Nathanael Harapan, Hyeon Joo Yoo

**Affiliations:** 1 Department of Neurosurgery, University Hospital, Ludwig-Maximilians-University of Munich, Marchionistr. 15, 81377 Munich, Germany; 2 Department of Neuropathology, Leipzig University Hospital, Leipzig, Germany; 3 Department of Radiotherapy and Radiation Oncology, University Hospital, Ludwig-Maximilians-University of Munich, Munich, Germany; 4 Department of Neurosurgery, Heidelberg University Hospital, Heidelberg, Germany

**Keywords:** COVID-19, SARS-CoV-2, Central nervous system, Neurological symptoms, Neurological manifestations, Neurological complications

## Abstract

Severe acute respiratory syndrome coronavirus 2 (SARS-CoV-2), a novel coronavirus, is responsible for the outbreak of coronavirus disease 19 (COVID-19) and was first identified in Wuhan, China in December 2019. It is evident that the COVID-19 pandemic has become a challenging world issue. Although most COVID-19 patients primarily develop respiratory symptoms, an increasing number of neurological symptoms and manifestations associated with COVID-19 have been observed. In this narrative review, we elaborate on proposed neurotropic mechanisms and various neurological symptoms, manifestations, and complications of COVID-19 reported in the present literature. For this purpose, a review of all current published literature (studies, case reports, case series, reviews, editorials, and other articles) was conducted and neurological sequelae of COVID-19 were summarized. Essential and common neurological symptoms including gustatory and olfactory dysfunctions, myalgia, headache, altered mental status, confusion, delirium, and dizziness are presented separately in sections. Moreover, neurological manifestations and complications that are of great concern such as stroke, cerebral (sinus) venous thrombosis, seizures, meningoencephalitis, Guillain–Barré syndrome, Miller Fisher syndrome, acute myelitis, and posterior reversible encephalopathy syndrome (PRES) are also addressed systematically. Future studies that examine the impact of neurological symptoms and manifestations on the course of the disease are needed to further clarify and assess the link between neurological complications and the clinical outcome of patients with COVID-19. To limit long-term consequences, it is crucial that healthcare professionals can early detect possible neurological symptoms and are well versed in the increasingly common neurological manifestations and complications of COVID-19.

## Introduction

On March 11, 2020, the World Health Organization (WHO) declared the coronavirus disease 2019 (COVID-19) to be a world pandemic. COVID-19 is caused by the recently identified severe acute respiratory distress syndrome coronavirus 2 (SARS-CoV-2) and is an ongoing global health emergency [1]. As of January 10, 2021, there are 90.08 million confirmed cases of COVID-19 globally in 218 countries and over 1.93 million deaths (https://www.worldometers.info/coronavirus/). Among all closed cases, 64.46 million subjects have recovered throughout the world, pointing to a recovery rate of around 97%.

Most patients with COVID-19 present with mild respiratory illness such as dry cough, fever, and dyspnea. However, various neurological manifestations have also been associated with COVID-19 at presentation or in the course of the disease [2]. At least one subjective neurological symptom has been reported in over 90% of patients with COVID-19, which highlights the importance of subsequent neurological implications of the disease [3]. Headache, confusion, and dizziness are the most common general non-specific neurological symptoms observed in COVID-19 patients [4]. Furthermore, numerous studies have reported neurological complications of SARS-CoV-2 infection that have a potentially detrimental effect on the outcome of patients with COVID-19 [5]. Based on the available literature, patients with severe COVID-19 infection tend to develop more neurological abnormalities in comparison to those with mild infection [6].

This narrative review aims to outline the current knowledge on existing neurological symptoms, manifestations, and complications in COVID-19 patients.

## Methods

We searched the PubMed database for articles in English, Spanish or German published until December 27, 2020. Different key words related to COVID-19 and neurological symptoms, manifestations and complications in titles and abstracts were used (“Neurology”, “Neurological symptoms”, “Neurological manifestations”, “Neurological complications”, “Neuro”, “COVID-19”, “SARS-CoV-2”, “Headache”, “Dizziness”, “Hyposmia”, “Anosmia”, “Meningitis”, “Encephalitis”, “Encephalopathy”, “Myalgia”, “Seizure”, “Epilepsy”, “Stroke”, “Ischemia”, “Ataxia”, “Myelitis”, “PRES”).

All relevant publications were independently screened by both authors (BNH and HJY) in their entirety to determine eligibility for inclusion. Apart from studies with a conclusive outcome, editorials, commentaries, case reports, case series, opinion letters, and viewpoints were considered in this review to fully exploit the potential offered by current literature. Exclusion was mainly based on topic, e.g. if the full text article did not primarily focus on a neurological symptom, manifestation, or complication of COVID-19. Results are reported in a narrative manner and divided into different sections within the text of this review.

## Results

### Proposed neurotropic mechanisms of COVID-19 leading to neuropathology

Moriguchi and colleagues first reported the presence of SARS-CoV-2 RNA in the cerebrospinal fluid (CSF) of a patient with encephalopathy and COVID-19 using real-time reverse transcription PCR (RT-PCR) [7]. Although further cases have since been reported [8–10], SARS-CoV-2 was not detected in the CSF of most COVID-19 patients with neurological manifestations [11, 12]. There are three theoretical explanations for this finding. First of all, the virus is mainly cell-bound and spreads from cell to cell without invading the CSF. Second, CSF sampling requires a certain limit of detection, which the virus barely reaches despite its presence. Finally, the presence of haem products in CSF could interfere with the role of polymerase for detection of SARS-CoV-2.

Neurological symptoms and manifestations can affect three different systems: the central nervous system (CNS), the peripheral nervous system (PNS), and the musculoskeletal system [13]. Several hypotheses describe the possible mechanisms of action causing the neurological symptoms of COVID-19. SARS-CoV-2 seems to have different mechanisms that allow the virus to enter and damage the CNS: systemic hematogenous spread or neuronal retrograde dissemination. These two potential neurotropic mechanisms of SARS-CoV-2 involve the compromise of the blood brain barrier causing neuronal cell death due to viremia on the one hand and the entry of SARS-CoV-2 through the olfactory bulb and subsequent transport to neurons of the brain on the other hand [14]. Although the neuroinvasiveness of SARS-CoV-2 has not yet been fully understood, multiple lines of evidence implicate that both hematogenous and neuronal route can be utilized to penetrate the CNS [15, 16].

The first mechanism relies on angiotensin-converting enzyme 2 (ACE 2) as the functional receptor for SARS-CoV-2. The virus’ spike protein interacts with ACE 2 receptors expressed in neurons and glial cells of the brain, which makes the brain susceptible for neuroinvasion. After binding to the ACE 2 receptor, the transmembrane protease serine 2 (TMPRSS2) leads to proteolytic cleavage and priming of the spike protein which allows the virus to gain entry into the host cells [16]. In humans, ACE 2 receptors are widely expressed in airway epithelia, kidney cells, small intestine, lung parenchyma, vascular endothelium and throughout the CNS including neurons, astrocytes, oligodendrocytes, substantia nigra, ventricles, middle temporal gyrus, posterior cingulate cortex and olfactory bulb [17]. SARS-CoV-2 has, therefore, the potential to infect neurons and glial cells expressed throughout the CNS.

After infecting the airways, SARS-CoV-2 can pass through the epithelial barrier and gain access to the bloodstream via infection of endothelial cells of the blood brain barrier or blood–CSF barrier in the choroid plexus. SARS-CoV-2 can, moreover, infect leukocytes, which disseminate towards other tissues in the human body and then cross the barrier to gain access to the CNS; referred to as a Trojan horse mechanism [16]. Leukocytes release pro-inflammatory cytokines including TNF, which damages oligodendrocytes and neurons. They produce chemokines such as CCL5, CXCL10, and CXCL11 that induce chemoattraction of activated T cells. Astrocytes release chemokines such as CCL2, CCL5 and CXCL12, which serve to recruit more infected leukocytes. On the whole, we think that many more factors are involved in the inflammatory process and that SARS-CoV-2 ultimately initiates a vicious circle of neuroinflammation [15].

The second neuropathological pathway describes the invasion of the brain via the olfactory bulb and the upper nasal transcribial route by which SARS-CoV-2 spreads transneuronally to different zones of the brain.

Notably, several viruses can also infect and migrate through peripheral nerves to reach the CNS [18]. Regardless of which transmission route the virus takes advantage of, once the virus reaches the CNS, neurotropism and the subsequent immune response will cause a CNS pathology, resulting in a disease. Due to the high expression of ACE 2 in the gastrointestinal system, a fecal–oral pathway has further been hypothesized as a potential transmission pathway of SARS-CoV-2 [19]. Moreover, indirect immune-mediated CNS damage through cytokine storm is another possible neuropathological mechanism observed in COVID-19 patients [20]. Present literature on neuropathological alterations associated with COVID-19 is scarce. Evidence for COVID-19 infection involving direct neurotropism is limited, whereas hypoxia-associated neuropathological features that suggest a defect in microcirculation has been shown in an autopsy study [21].

### Neurological symptoms

Interestingly, neurological symptoms and conditions may precede typical respiratory symptoms by several days or were even the only indicators of SARS-CoV-2 infection in otherwise asymptomatic COVID-19 carriers. Due to limited data, this information cannot be specified for each symptom/manifestation separately.

Current literature suggests that neurological symptoms and manifestations resulting from COVID-19 can occur prior, during, and even after respiratory involvement [22].

#### Gustatory and olfactory dysfunctions/taste and smell (hyposmia/anosmia) impairment

Gustatory and olfactory disorders are the most common sudden neurologic symptoms of COVID-19 associated with the involvement of the PNS, which seem to develop in the early stages of the disease and is thus considered as useful diagnostic markers [23].

Preliminary reports implicate that olfactory sensory neurons do not express ACE 2, which prevent SARS-CoV-2 from infecting these cells. However, cells in the olfactory epithelium do express ACE 2 and are consequently vulnerable to SARS-CoV-2 infection. Damage to the olfactory epithelium rather than neuronal injury seems to be the cause of anosmia [24, 25].

In a systematic meta-analysis, the pooled prevalence for taste disturbances has been estimated at around 38.5% and 35.8% for smell disturbances [26]. Of note, several studies report a much higher incidence of taste and smell disturbances. For instance, out of 417 COVID-19 patients who participated in a multicenter European study, 85.6% had decreased smell function related to the infection, while 88.0% of the patients reported gustatory disorders characterized by impairment of different taste modalities. A significant positive association between olfactory and gustatory dysfunctions has been observed. The same study hints at a recovery rate of approximately 25.5% for both olfactory and gustatory functions throughout the 2 weeks after the resolution of typical respiratory symptoms [27].

In a study of Qiu et al., 10% of COVID-19 patients had only olfactory or gustatory symptoms, and 19% had olfactory and/or gustatory complaints prior to any other COVID-19 symptom.

Moreover, 25% of children had only olfactory or gustatory symptoms at COVID-19 presentation, indicating that olfactory or gustatory disorders are possible early identifiers of COVID-19 and, therefore, has important implications for patient screening and disease control [28]. Further evaluation of olfactory function in COVID-19 patients that indicate a high prevalence of olfactory disorders has been carried out on hospitalized and non-hospitalized patients [29, 30]. Unsurprisingly, olfactory dysfunction has been suggested as a biomarker for COVID-19 and smell testing may help the early detection of COVID-19 patients who need early treatment or quarantine as a part of preventive measures aimed at controlling the spread of COVID-19 [31].

#### Myalgia

Myalgia is a common symptom observed in COVID-19 patients. The prevalence of myalgia greatly varies between studies, ranging from 3.36% to over 64% [32] with an estimated pooled prevalence of around 19.3% [26]. It has been proposed that generalized inflammation and cytokine storm could be the pathophysiological background of myalgia [33]. At present, it is still uncertain whether muscle manifestation of COVID-19 is due to an unspecific systemic inflammation or direct muscle invasion. Elevated levels of lactate dehydrogenase and creatine kinase can be observed in patients with severe COVID-19 disease. Interestingly, present literature suggests that myalgia is not necessarily related to severe cases of COVID-19 and its mere presence is, therefore, not a reliable prognostic factor for severe COVID-19 disease [34].

#### Headache

Headache has been reported to be the most common non-specific neurological symptom in a series of studies. The prevalence of headache varies depending on the studies’ number of cases. For instance, some authors observe that around 6% [35, 36] and 8% [37] of all COVID-19 patients presented with headache, while others propose a much higher prevalence at around 20% [38] and 25% [39]. 14.7% is the estimated pooled prevalence of this symptom [26]. Headache was frequently observed at the time of admission. Studies suggest that after fever, cough, myalgia/fatigue and dyspnea, headache is the fifth most frequent symptom of COVID-19 [37, 40, 41]. A personal clinical case written from the perspective of a neurologist, specifically a headache expert, shows that the headache associated with COVID-19 can be classified into two phases. While the headache in the initial phase, among others, included a diffuse pain, moderate in intensity that is attributed to systemic viral infection, the headache after 7–10 days was accompanied by photophobia and neck stiffness and is provoked by cytokine storm [42]. Other authors reported moderate–severe bilateral headache with pulsating or pressing quality in the temporoparietal, forehead, or periorbital region which is exacerbated when bending over [43]. As a non-specific symptom, headache might be interpreted as a random association to COVID-19; however, there is clear evidence that COVID-19 patients who had never suffered from recurrent headache before, suddenly experience persistent disabling daily headache due to SARS-CoV-2 infection [44]. The onset of a new daily headache in subjects without previous personal headache history suggests a causal relation between headache and COVID-19 with severe pain more often presenting as a prodromal symptom. Headache has further been described as the first COVID-19 symptom in some cases [45].

#### Altered mental status/confusion/delirium

A case study from England indicates that acute confusion/delirium can be a primary manifestation and the only presenting symptom of COVID-19 without overt respiratory symptoms [46]. A Chinese study estimates the prevalence of confusion at around 9% [4], which coincides with the pooled prevalence of disturbances in consciousness/altered mental status of around 9.6% [26].

Primary neuroinvasive hypotheses include the ability of SARS-CoV-2 to access the CNS directly via invasion of the olfactory bulb and secondary systemic mechanisms such as inflammatory cytokines, hypoxemia, and oxidative stress that are caused by ARDS (acute respiratory distress syndrome). These result in primary and secondary encephalopathy, respectively.

Other authors acknowledge delirium as a complication of COVID-19 and state that the presence of comorbidities found during the SARS-CoV-2 infection may facilitate the onset of an acute confusional state [47]. Independent of the etiology of delirium, hospitals should consider adding mental status changes to the list of testing criteria since delirium may be the only presenting symptom [48].

#### Dizziness

The prevalence of dizziness is estimated at around 8% [49] to 9% [50], which corresponds to the overall pooled prevalence of dizziness at 8.77% in a systemic review regarding neurological characteristics of COVID-19 patients [51]. A retrospective, observational case series even describes dizziness to be the leading neurologic finding even before headache [6]. Of note, a case report shows a COVID-19 patient with a sudden onset of dizziness and dry throat with no other accompanying typical symptoms such as fever, cough, and headache [52]. Although dizziness seems to be unrelated to COVID-19 at first sight, clinicians should be vigilant about this neurological symptom as it may be caused by COVID-19, especially in the absence of common respiratory symptoms.

### Neurological manifestations and complications

Among the studies conducted within a hospital network in Chicago, Illinois, neurologic manifestations were identified in about 42% at COVID-19 onset, in 63% at hospitalization and in 82% at any time during the course of the disease [53]. Similar findings were obtained in 36% of hospitalized COVID-19 patients in China and in around 60% of COVID-19 patients in Europe [6, 54]. The differences in frequencies seem to rely on genetic factors such as ACE 2 receptor polymorphism as well as SARS-CoV-2 strain variations [55]. Recent literature indicates higher rates of mortality in COVID-19 patients with pre-existing chronic neurological disorders [56].

Biological and clinical observations suggest that SARS-CoV-2 may be responsible for many neurological manifestations, which can be spilt into three main categories based on the following presumed underlying mechanisms. The first mechanism includes neurological consequences of pulmonary disease and associated systemic disease such as systemic inflammatory response syndrome, sepsis, and multi-organ failure. Encephalopathy and stroke can be assigned to this category. The second mechanism involves neurological manifestations that result from direct invasion of the CNS by the virus (e.g. encephalitis). Post-infectious, immune-mediated complications such as GBS and its variants represent the last mechanism. As SARS-CoV-2 continues to spread across the world, our knowledge and understanding of neurological manifestations in patients is constantly evolving.

#### Cerebrovascular diseases

##### Stroke

The acute ischemic stroke incidence among COVID-19 patients is estimated at 2.3% with a pooled prevalence for acute cerebrovascular disease of 2.6% [26]; the prevalence of acute cerebrovascular diseases in hospitalized COVID-19 patients with more severe infection almost reaches 6% [6]. Most of these cases presented with arterial stroke. A possible explanation for the occurrence of coagulation disorders in COVID-19 patients is the enhanced thrombus formation under conditions of hypoxia.

SARS-CoV-2 can infect endothelial cells resulting in endothelial dysfunction and arterial as well as venous micro- and macrovascular complications [57, 58]. Local inflammation and a vasculitic process in cerebral arterial walls are induced. Inflammation and apoptosis of endothelial cells after SARS-CoV-2 infection has recently been reported at autopsy in the lung, kidney, heart, and bowel [59]. The permeability of blood brain barrier might be increased via inflammatory cells accumulating in the vascular wall [57].

Viral infections are believed to stimulate and initiate a coagulation cascade, while complex cross-reactions occur between coagulative hemostasis and inflammation. COVID-19 particularly causes sepsis-induced coagulopathy, which is characterized by elevated prothrombin time, elevated levels of d-dimer and thrombocytopenia without hypofibrinogenemia [60]. A downregulation of natural anticoagulant mechanisms due to inflammatory mediators and disruption of the coagulation system is involved in the pathophysiology. Generally, hypercoagulability is a major contributor to COVID-19-related complications and can be the cause of consecutive thromboembolic events in both the arterial and venous vascular beds [61]. Consequently, it is reasonable to perform laboratory monitoring of coagulation markers such as fibrinogen levels, D-dimer and markers of inflammation such as CRP and IL-6 levels to determine an underlying prothrombotic or inflammatory response, potentially helping to guide treatment [62]. Despite thromboprophylaxis, there seems to be a high cumulative incidence of thrombotic complications in critically ill COVID-19 patients, especially in those with pneumonia [63]. On the other hand, several findings suggest that ischemic cerebrovascular disease may simultaneously develop in the course of COVID-19 independently of a critical disease process [64].

There is a hint that COVID-19 patients have more severe anterior circulation large vessel occlusion strokes with a higher rate of multivessel occlusion and higher infarct core volume than control patients [65]. Thus, this neurological complication provides an explanation why COVID-19 leads to very poor outcome in some individuals [66, 67].

##### Cerebral venous (sinus) thrombosis

Although an underestimation cannot be excluded, cerebral venous thrombosis is a rather rare complication (pooled prevalence of 0.3% [26]). Patients without any antecedent risk factors for cerebral venous thrombosis may develop this neurological sequela due to the prothrombotic state triggered by COVID-19 [68–70]. Initial symptoms may include signs of increased intracranial pressure (ICP) such as progressive headache, visual problems, papilledema, focal neurologic deficits, decreased consciousness, and seizures [71]. The diagnosis of deep cerebral vein thrombosis can even be complicated by hemorrhagic venous infarction with large necrotic areas. Cerebral venous sinus thrombosis is primarily diagnosed based on clinical and radiological criteria with magnetic resonance imaging (MRI) and venography and computed tomographic venography being the non-invasive imaging modalities of choice [72]. Treatment with heparin anticoagulation is favored, either with therapeutic doses of low molecular weight heparin (LMWH) or unfractionated heparin (UFH). Based on currently available evidence, LMWH seems to be more effective than UFH with lower rates of mortality, and hence LMWH is recommended as the first-line therapy if there is no other contraindication [73, 74].

#### Epilepsy and seizures

With increasing case reports regarding COVID-19 patients presenting with seizures, current literature suggests that COVID-19 can reduce seizure threshold in patients with an existing seizure disorder. What is more, COVID-19 does not only worsen seizure control in patients with previously well-controlled seizures, but it can trigger new-onset seizures in patients with no known history of seizures [75–79]. Direct effects of COVID-19 on patients with epilepsy and the exact prevalence of new-onset epilepsy cases is still unclear (estimated pooled prevalence 0.9% [26]). Hypoxia, fever, multi-organ failure, and severe metabolic or electrolyte disarrangements due to COVID-19 can possibly trigger seizures [80]. Seizure can also occur secondary to a meningitis/encephalitis associated with SARS-CoV-2 infection [7]. Furthermore, seizures can be the initial presentation of COVID-19 and patients may have no respiratory symptoms at all [81]. Apart from generalized tonic–clonic seizures, focal seizures have also been observed as a clinical feature of COVID-19 [82]. Pediatric seizure cases have been reported and therefore, seizure must be recognized as a potential COVID-19 presentation in the pediatric age groups, even in children without fever (afebrile seizures) [83, 84]. In addition to antiepileptic drugs, patients who severely suffer from COVID-19 and further demonstrate clinical signs of seizures or encephalopathy could benefit from electroencephalography monitoring to early diagnose and treat seizures [85]. Despite the lack of clear evidence suggesting an additional risk of symptomatic seizures in COVID-19 patients [86], uncommon and atypical clinal presentation of COVID-19 patients in association with seizure must be considered to prevent complications and long-term sequelae.

#### Meningitis, encephalitis, and meningoencephalitis

Several case reports’ findings of patients with COVID-19 are suggestive of meningitis and encephalitis. COVID-19 patients can initially complain of headache, fever, and a new-onset seizure [7, 87]. In some COVID-19 patients, the presence of SARS-CoV-2 was found in the CSF, confirming that this neurological manifestation is to be attributed to the virus [7, 9, 88]. However, COVID-19 patients presenting with acute meningoencephalitis with neither SARS-CoV-2 nor other viral pathogens detected in the CSF have also been observed [89, 90]. Therefore, undetectable SARS-CoV-2-RNA in the CSF may indicate that other than direct brain infection might be responsible for meningoencephalitis, e.g. peri-infectious inflammation and altered neurotransmission. COVID-19 patients’ CSF may reveal lymphocytic pleocytosis [87, 89]. Since meningoencephalitis can be complicated by intracerebral und subdural hematomas, early detection is essential to provide appropriate treatment and prevent hemorrhagic encephalopathy that can lead to severe invalidity or threaten the patient’s life. Due to the detrimental consequences, physicians should always be aware of this possible manifestation [91].

#### Guillain–Barré syndrome (GBS)

As an inflammatory polyradiculoneuropathy associated with numerous infections (e.g. Campylobacter jejuni, Epstein–Barr virus, cytomegalovirus), COVID-19 patients with GBS can present with various neurological symptoms. For instance, lower limb weakness and paresthesia that may lead to generalized tetraparesis or tetraplegia evolving over a period of several days have been repeatedly observed [92]. Typically, MRI performed with the administration of gadolinium reveals enhancement of the cauda equina nerve roots on postcontrast findings and asymmetrical thickening and hyperintensity of postganglionic roots of the brachial and lumbar plexuses [93, 94]. Moreover, cytoalbuminologic dissociation of the CSF is a characteristic finding in GBS, pointing to nerve root involvement. Analysis of CSF usually reveals normal white cell count and increased protein concentrations [95, 96]. Results of electrophysiological studies are frequently consistent with a demyelinating polyradiculoneuropathy and/or axonal damage. Of note, it seems that most COVID-19 patients who suffer from GBS predominantly presented with a demyelinating electrophysiological subtype (acute inflammatory demyelinating polyneuropathy) [97, 98]. Generally, GBS shows a good response to intravenous immunoglobulin treatment with significant neurological improvement, suggesting an immune-mediated nature of neuropathy [99, 100].

#### Miller Fisher syndrome (MFS)

MFS is classified as a variant of GBS and characterized by a triad of ophthalmoplegia, ataxia, and areflexia [101]. Comparable to GBS, symptoms may be preceded by a viral infection. This neurological complication is frequently associated with anti-GQ1b antibodies detected in serum samples from MFS patients, although several case reports noted negative antiganglioside antibodies [102–104]. Negative antibody testing in COVID-19 patients with MFS may signal that symptoms are caused by viral neurotropism and not immune-mediated injury [105]. Similar to the treatment of GBS, intravenous immunoglobulin usually results in subsequent improvement of neurologic symptoms [104, 106].

#### Acute myelitis

Several cases of COVID-19 patients with transverse myelitis have been observed during the ongoing COVID-19 pandemic. Paresthesia and hypoesthesia of the feet progressing to the abdominal area may be the first symptoms of myelitis. This can result in weakness of the lower limbs that can rapidly progress to paraplegia with total anesthesia below a certain spinal cord level and eventually sphincter incontinence [107]. Apart from transverse myelitis that is caused by COVID-19, the differential diagnosis of acute-onset ascending symmetric paraplegia that must be considered include acute inflammatory demyelinating polyradiculoneuropathy (GBS), traumatic spinal injury, epidural hematoma and/or abscess, post-diphtheric polyneuropathy, acute intermittent porphyria, periodic paralysis and paralytic poliomyelitis [108]. An aberrant immune response and immune-mediated pathogenesis are possible explanations for myelitis in COVID-19 patients. MRI is the most frequently used imaging modality for the diagnosis of suspected cases of acute myelitis since it does not only indicate spinal cord lesions but also excludes other possible pathologies that may present with similar clinical symptoms [109]. Neurologic improvement can be achieved through immunomodulatory treatment such as steroids and plasmapheresis [110]. For instance, corticosteroid therapy such as intravenous methylprednisolone 1 g per day for 3 days may rapidly improve neurological symptoms [111].

#### Posterior reversible encephalopathy syndrome (PRES)

Clinical features of this disease can, among others, include non-specific symptoms such as headache, altered consciousness, visual disturbances, seizures, and fluctuations in blood pressure. PRES may result from elevated inflammatory markers and cytokines leading to changes in endothelial morphology, impairment of the blood–brain barrier and consecutive increased vascular permeability [112]. MRI of the brain typically indicates cerebral vasogenic edema in the parietal and occipital regions bilaterally, occasionally associated with hemorrhagic lesions [113]. Patients with severe SARS-CoV-2 infection have a drastic inflammatory reaction, provoking a cytokine storm which damages the blood brain barrier and resulting in PRES. The associated hemorrhage seen in several COVID-19 patients [114, 115] can be explained by coagulopathy and both thrombotic and hemorrhagic pathologies of severe SARS-CoV-2 infection. As a rather rare neurological condition, it seems unlikely that the occurrence of this disorder in COVID-19 patients is completely unrelated to SARS-CoV-2 infection, especially since there are signs of increased cases of PRES in the context of the COVID-19 pandemic (e.g. higher weekly average cases of PRES than over the past 5 years [116]).

(Table 1 is a summary on neurological symptoms, manifestations, and complications mentioned in the present literature, including those with scarce detailed report or insufficient data).

**Table 1:** Neurological symptoms, manifestations, and complications in association with COVID-19

Neurological symptoms	Neurological manifestations and complications
Gustatory dysfunctions (38.5%)	Stroke (2.3%)
Olfactory dysfunctions (hyposmia/anosmia) (35.8%)	Epilepsy and seizures (0.9%)
Myalgia (19.3%)	Cerebral venous (sinus) thrombosis (0.3%)
Headache (14.7%)	Meningitis, encephalitis, meningoencephalitis
Altered mental status (9.4%) [5, 117]	Guillan–Barré syndrome
Dizziness (8.77%)	Miller Fisher syndrome/Bickerstaff’s encephalitis [118]
Nausea and vomiting (4.6%) [51, 119]	Acute myelitis
Neuralgia (2.3%) [32, 120]	Posterior reversible encephalopathy syndrome (PRES)
Ataxia (0.3%) [121, 122]	Acute hemorrhagic necrotizing encephalopathy [123, 124]
Myoclonus [125, 126]	Acute demyelinating encephalomyelitis (ADEM)-like pathology [127, 128]
Diplopia [129]	Posthypoxic necrotizing leukoencephalopathy [130]
Vision loss [131]	CNS vasculitis [117]
Stupor [2]	Acute cerebellitis [132]
Meningism [133]	Movement disorders [134]
Dysexecutive syndrome [135]	Intensive-care-unit acquired neuropathy [2]
Bilateral leg stiffening [136]	Rhabdomyolysis [137]
Sustained upward gaze [136]	Critical illness myopathy [138, 139]
	Necrotizing autoimmune myositis (NAM) [140]
	Acute mesenteric ischemia [141]

This table summarizes different neurological symptoms, manifestations, and complications observed in association with COVID-19. Symptoms, manifestations, and complications written in bold are each addressed separately in the manuscript. All other symptoms, manifestations, and complications have been reported or at least mentioned in articles regarding COVID-19; however, insufficient data restrict detailed information.

The percentage in parentheses indicates the prevalence of the neurological symptom/manifestation/complication.

## Conclusions

At present, most publications focus on the general clinical presentation and common respiratory aspects of COVID-19, whereas our review highlights the nervous system involvement of SARS-CoV-2 infection with respect to its diverse neurological presentation. Since most reports and studies mainly put emphasis on respiratory symptoms, the prevalence of neurological sequalae of COVID-19 might be underestimated. Many neurological symptoms and manifestations have been reported in several COVID-19 cases, however, insufficient data restricts detailed description of these symptoms’ prevalence and characteristics. From the available data, we conclude that non-specific neurological symptoms may indicate a SARS-CoV-2 infection and thus, clinicians should always be vigilant for neurological manifestations and detect them at an early stage to prevent inappropriate management of COVID-19 patients and address neurological complications adequately. Noteworthily, patients with severe COVID-19 infection are more prone to develop neurological complications than nonsevere patients.

We hope that this review article allows healthcare professionals to be aware of the heterogenous neurological symptoms and manifestations of COVID-19 when dealing with the current viral pandemic. Clinicians must be alert to potential neurological complications during the diagnosis and treatment of patients with COVID-19 to reduce patient morbidity and mortality. Further studies are warranted to identify the broad spectrum of neurological symptoms, manifestations, and complications of COVID-19 and the underlying pathophysiological mechanisms.

## Data Availability

Authors can confirm that all relevant data are included in the article. Dataset(s) derived from public resources and made available with the article (references).
